# Orexin A Enhances Pro-Opiomelanocortin Transcription Regulated by BMP-4 in Mouse Corticotrope AtT20 Cells

**DOI:** 10.3390/ijms22094553

**Published:** 2021-04-27

**Authors:** Satoshi Fujisawa, Motoshi Komatsubara, Naoko Tsukamoto-Yamauchi, Nahoko Iwata, Takahiro Nada, Jun Wada, Fumio Otsuka

**Affiliations:** 1 Department of Nephrology, Rheumatology, Endocrinology and Metabolism, Okayama University Graduate School of Medicine, Dentistry and Pharmaceutical Sciences, 2-5-1 Shikata-cho, Kitaku, Okayama 700-8558, Japan; fujisawa_sa@s.okayama-u.ac.jp (S.F.); swe_etfish@yahoo.co.jp (M.K.); n-tsukamoto@okayama-u.ac.jp (N.T.-Y.); junwada@okayama-u.ac.jp (J.W.); 2 Department of General Medicine, Okayama University Graduate School of Medicine, Dentistry and Pharmaceutical Sciences, 2-5-1 Shikata-cho, Kitaku, Okayama 700-8558, Japan; nao53mayflower@gmail.com (N.I.); takahironada@okayama-u.ac.jp (T.N.)

**Keywords:** anterior pituitary, bone morphogenetic protein (BMP), corticotrope, orexin, pro-opiomelanocortin (POMC)

## Abstract

Orexin is expressed mainly in the hypothalamus and is known to activate the hypothalamic–pituitary–adrenal (HPA) axis that is involved in various stress responses and its resilience. However, the effects of orexin on the endocrine function of pituitary corticotrope cells remain unclear. In this study, we investigated the roles of orexin A in pro-opiomelanocortin (POMC) transcription using mouse corticotrope AtT20 cells, focusing on the bone morphogenetic protein (BMP) system expressed in the pituitary. Regarding the receptors for orexin, type 2 (OXR2) rather than type 1 (OX1R) receptor mRNA was predominantly expressed in AtT20 cells. It was found that orexin A treatment enhanced POMC expression, induced by corticotropin-releasing hormone (CRH) stimulation through upregulation of CRH receptor type-1 (CRHR1). Orexin A had no direct effect on the POMC transcription suppressed by BMP-4 treatment, whereas it suppressed Smad1/5/9 phosphorylation and Id-1 mRNA expression induced by BMP-4. It was further revealed that orexin A had no significant effect on the expression levels of type I and II BMP receptors but upregulated inhibitory Smad6/7 mRNA and protein levels in AtT20 cells. The results demonstrated that orexin A upregulated CRHR signaling and downregulated BMP-Smad signaling, leading to an enhancement of POMC transcription by corticotrope cells.

## 1. Introduction

Orexins are neuropeptides that are expressed primarily in the hypothalamus and are produced in two isoforms, orexin A (ORX) and orexin B, from a common precursor protein, prepro-orexin [1]. There are two types of G protein-coupled receptors, named orexin type 1 (OX1R) and type 2 (OX2R) receptors. Orexin B binds only to OX2R, while orexin A binds to both OX1R and OX2R [2]. Since its discovery in 1998, orexin has been reported to have various effects on sleep–wake regulation, feeding behavior, emotions, and the autonomic nervous system involved in stress control [1,3,4]. Orexin is also known to have physiological effects in peripheral tissues, including the endocrine system [5].

An interaction between the hypothalamic–pituitary–adrenal (HPA) system in response to stressful stimuli and orexin neurons has been recognized. Orexin has been reported to enhance adrenocortical hormone secretion [6,7] in the HPA axis, and orexin neurons are further activated by corticotropin-releasing hormone (CRH) [8]. Previous studies showed that an intracerebroventricular injection of orexin increased adrenocorticotropin (ACTH) and corticosterone levels in the blood, which were reversed by CRH receptor inhibitors [9,10,11]. Moreover, that orexin increases CRH and arginine vasopressin (AVP) mRNA expression in the paraventricular nucleus (PVN) of the hypothalamus [12]. These results suggested that orexin plays an important role in regulating the HPA axis, which is involved in stress responses, mainly in the hypothalamus. Moreover, in the adrenal cortex, it was shown that orexin A rather than orexin B directly stimulates corticosterone secretion in adrenal cortical cells and that this effect is not blunted by ACTH receptor antagonists [13,14]. Those studies showed that orexin has a functional role in the adrenal cortex, independent of ACTH-stimulated control.

ACTH is produced from the precursor protein pro-opiomelanocortin (POMC), which is synthesized in corticotrope cells. POMC is also cleaved into multiple peptide hormones in the pituitary and hypothalamus. POMC neurons are located in the arcuate nucleus of the hypothalamus; they suppress appetite, regulate energy balances, and are thought to be involved in the mechanism of orexin-mediated feeding behavior [15]. Increasing evidence has shown a mutual relationship between orexin, the HPA axis, and POMC in the hypothalamus. Given the finding that OX2R was expressed in human corticotrope cells, orexin might be directly involved in the mechanism of hormone production by the anterior pituitary [16].

There has been growing evidence that BMPs, members of the transforming growth factor (TGF)β superfamily of proteins, play functional roles in various endocrine organs in an autocrine or paracrine manner [17]. In pituitary corticotrope AtT20 cells, BMP-4 is known to inhibit the tumorigenesis of corticotrope tumor cells [18,19]. We previously reported that BMP-4 was involved in the suppression of ACTH secretion by somatostatin receptor stimulation and also suppressed GHRP-2-induced ACTH secretion in AtT20 cells [20,21]. Interestingly, we recently revealed that orexin inhibits prolactin secretion by suppressing BMP signaling in rat somatolactotrope GH3 cells [22].

In the present study, we investigated the role of orexin in endocrine regulation in the pituitary gland, focusing on the relationship of orexin with BMP signaling by using the mouse pituitary corticotrope tumor cell line AtT20.

## 2. Results

**Figure 1:**
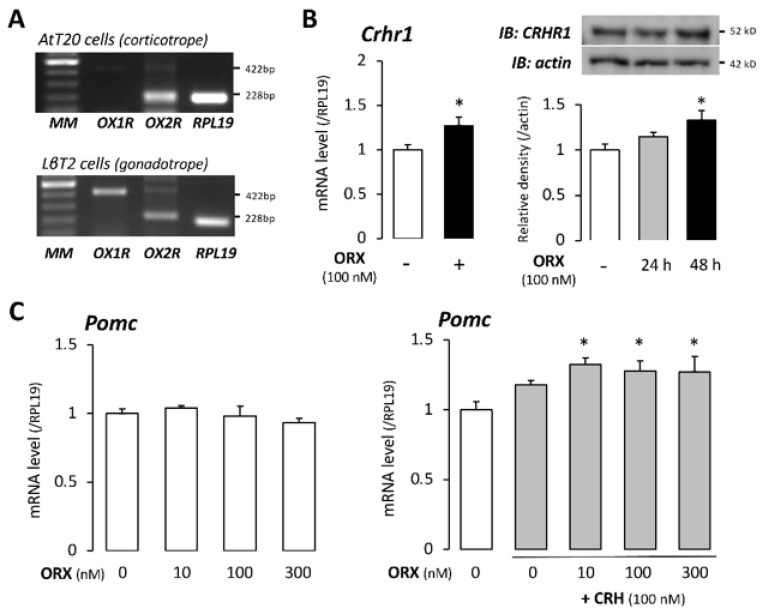
Expression of orexin receptors and the effect of orexin A on CRH receptor and POMC expression in AtT20 cells. (**A**) The expression of mRNAs encoding OX1R, OX2R, and RPL19 was examined by RT−PCR in AtT20 cells compared with that in mouse gonadotrope LβT2 cells. MM: molecular weight marker. (**B**) After preculture, cells were treated with orexin (ORX) in serum-free media. After 24−h culture, total cellular RNA was extracted, and after 24−h or 48−h culture, cell lysates were extracted. The mRNA expression levels of Crhr1 were quantified by qPCR, and the expression levels of target genes were standardized by the RPL19 level in each sample. The cell lysates were analyzed by immunoblotting (IB) with anti−CRHR1 and anti-actin. (**C**) After preculture, cells were treated with ORX alone or with the combination of ORX and CRH in serum-free media. After 24−h culture, total cellular RNA was extracted, and the mRNA expression levels of POMC were quantified by qPCR. The expression levels of target genes were standardized by the RPL19 level in each sample. Results are shown as means ± SEM of data from at least three independent experiments. Statistical analysis was performed by the unpaired *t*−test (**B**) or ANOVA (**B**,**C**). * *p* < 0.05 vs. between the control groups.

First, we evaluated the expression of orexin receptors in AtT20 cells. Although the expression of both OX1R and OX2R was detected in mouse pituitary gonadotrope LβT2 cells by RT-PCR, AtT20 cells mainly expressed OX2R, as shown in Figure 1A. AtT20 cells express CRHR1 as a receptor for CRH, and orexin A (100 nM) treatment enhanced the mRNA expression of CRHR1. To determine whether this orexin-induced change in the CRH receptor was also detectable at protein levels, Western blotting was performed. As shown in Figure 1B, orexin A (100 nM) treatment for 48 h increased the protein expression level of CRHR1 in a time-dependent manner. Therefore, we examined the function of orexin in CRH signaling in AtT-20 cells. Treatment with orexin A (10–300 nM) did not change basal mRNA levels of POMC for 24 h. However, in the presence of CRH (100 nM), co-treatment with orexin (10–300 nM) for 24 h enhanced CRH-induced mRNA expression of POMC (Figure 1C).

**Figure 2:**
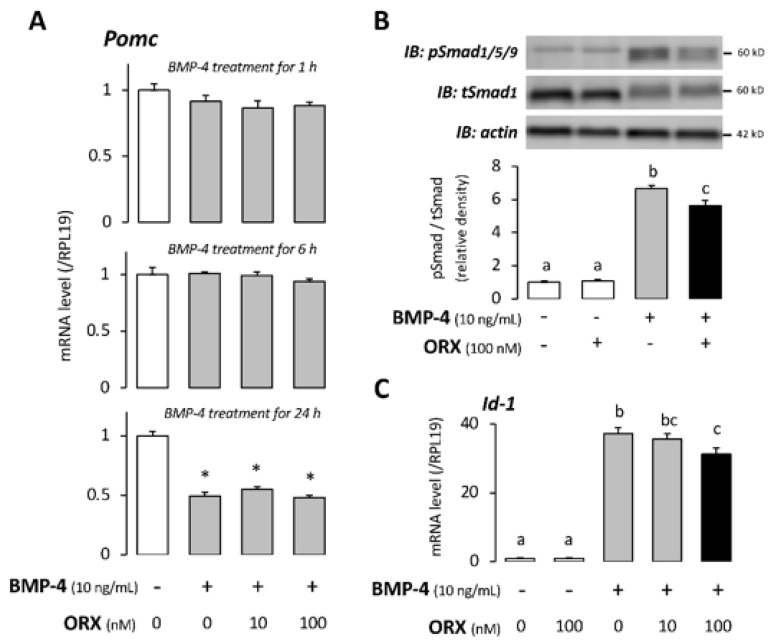
Effects of orexin A on BMP−receptor signaling in AtT20 cells. (**A**) After preculture, AtT20 cells were treated with the combination of orexin (ORX) and BMP−4 in serum−free media. After 24−h stimulation with orexin and 1−, 6−, and 24−h stimulations with BMP−4, total cellular RNA was extracted, and the mRNA expression levels of POMC were quantified by qPCR. The expression levels of target genes were standardized by the RPL19 level in each sample. (**B**) Cells were pretreated with ORX in serum−free media for 24 h. After 60-min stimulation with BMP−4, the cell lysates were subjected to immunoblot (IB) analysis using anti−pSmad1/5/9, anti−tSmad1, and anti−actin. (**C**) Cells were pretreated with ORX in serum−free media for 24 h. After 60−min stimulation with BMP−4, total cellular RNA was extracted, and mRNA levels of Id−1 were examined by qPCR. The expression levels of target genes were standardized by the RPL19 level in each sample. Results are shown as means ±SEM of data from at least three independent experiments. Statistical analysis was performed by ANOVA. Values with different superscript letters are significantly different at *p* < 0.05 (**B**,**C**). * *p* < 0.05 vs. between the control groups (**A**).

Next, we examined the effect of orexin on the action of BMP-4 in AtT-20 cells. We previously reported that BMP-4 suppressed POMC mRNA expression in AtT20 cells [20]. As shown in Figure 2A, BMP-4 (10 ng/mL) treatment for 24 h decreased POMC mRNA, consistent with the results of our previous study. Co-treatment with orexin (10–100 nM) did not change the POMC mRNA expression induced by BMP-4 treatment from 1 h to 24 h (Figure 2A). On the other hand, focusing on the effect of orexin on BMP-Smad signaling, stimulation with BMP-4 (10 ng/mL) for 1 h activated Smad1/5/9 phosphorylation in AtT20 cells (Figure 2B). Importantly, orexin A (100 nM) pretreatment for 24 h inhibited the phosphorylation of Smad1/5/9 by BMP-4 (Figure 2B). We next examined the effect of orexin A on the transcription of Id-1, a target gene for BMP-receptor signaling. As shown in Figure 2C, BMP-4 (10 ng/mL) treatment for 1 h significantly upregulated Id-1 mRNA, and co-treatment with orexin A (100 nM) suppressed the expression of Id-1 mRNA induced by BMP-4.

**Figure 3:**
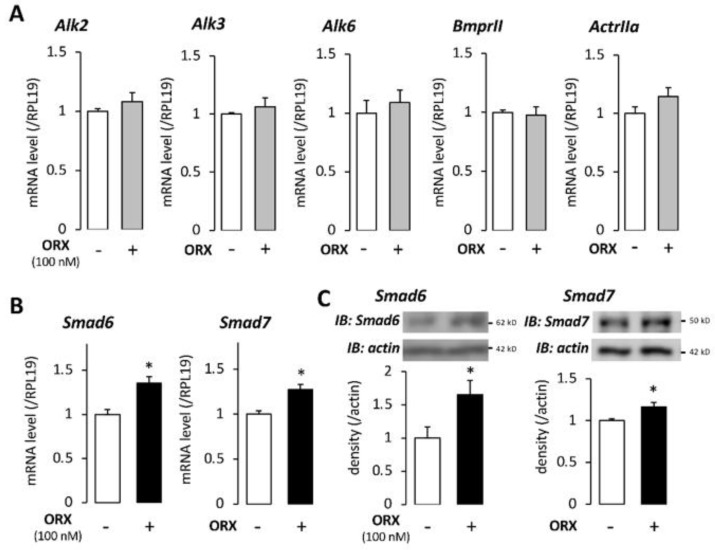
Effects of orexin A on the expression of BMP-receptor signaling molecules in AtT20 cells. (**A**,**B**) AtT20 cells treated with orexin (ORX) for 24 h were extracted, and the mRNA expression levels of Alk2, Alk3, Alk6, BmprII, ActrIIa (**A**), Smad6, and Smad7 (**B**) were quantified by qPCR. The expression levels of target genes were standardized by the RPL19 level in each sample. (**C**) Cells were treated with ORX in serum−free media for 24 h. The cell lysates were subjected to immunoblot (IB) analysis using antibodies that detect Smad6, Smad7, and actin. Results are shown as means ±SEM of data from at least three independent experiments. Statistical analysis was performed by the unpaired *t*-test. * *p* < 0.05 vs. between the control groups.

To elucidate the mechanism by which orexin A inhibits BMP-Smad signaling, we further examined the expression levels of BMP-receptor components using real-time PCR. As shown in Figure 3A, the expression levels of both BMP type I receptors (ALK2, ALK3, and ALK6) and type II receptors (BMPRIII and ACTRIIA) were unchanged after 24 h of treatment with orexin A (100 nM). Next, we examined the effect of orexin A on inhibitory Smads. Both Smad6 and Smad7 are known as inhibitory Smads, and they interfere with the receptor-regulated phosphorylation of other Smad molecules. Treatment with orexin A (100 nM) for 24 h enhanced the mRNA expression of both inhibitory Smad6 and Smad7 (Figure 3B). To examine whether the changes in the inhibitory Smads can be detected at the protein level, Western blot analysis was also performed. As a result, treatment with orexin A (100 nM) for 24 h significantly increased the protein expression levels of Smad6 and Smad7 (Figure 3C). These findings suggested that orexin A was involved in the BMP-Smad signaling in AtT20 cells through the upregulation of inhibitory Smad6/7.

## 3. Discussion

**Figure 4:**
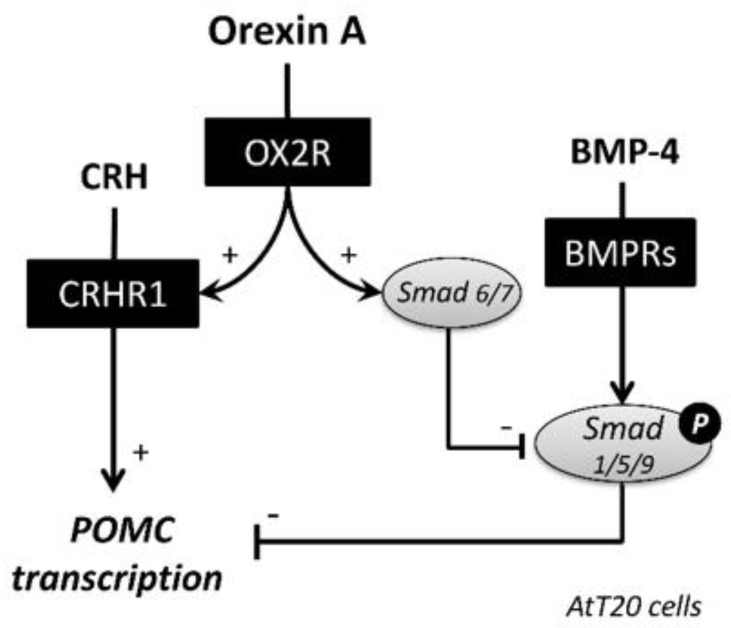
Functional roles of orexin A in the activities of CRH and BMP−4 in corticotrope cells. OX2R rather than OX1R was predominantly expressed in corticotrope AtT20 cells. Orexin A upregulated CRHR1 expression and CRH−induced POMC transcription. Orexin A also suppressed BMP−4−induced Smad1/5/9 phosphorylation and Id−1 transcription by upregulating inhibitory Smad6 and Smad7. Thus, orexin A has an enhancing effect on POMC expression by upregulating CRH signaling and downregulating BMP−Smad action, which is possibly involved in the control of stress responses and their resilience.

In the present study, we investigated the functional roles of orexin in CRH and BMP signaling by AtT20 cells. As for the orexin receptors, OX2R rather than OX1R was predominantly expressed in AtT20 cells. Orexin A enhanced CRHR1 expression and increased POMC mRNA expression in the presence of CRH. Regarding the BMP receptor signaling, orexin A treatment suppressed BMP-4-induced Smad1/5/9 signaling and Id-1 mRNA expression by AtT20 cells. It was also revealed that the BMP signaling was modulated by orexin A through the upregulation of inhibitory Smad6/7 expression. These results indicate that orexin affects the transcriptional regulation of POMC through CRH- and BMP-Smad signaling in corticotrope cells (Figure 4).

It has been reported that orexin is closely related to the regulation of the HPA axis. OX1R is widely distributed in the hypothalamus, while OX2R, but not OX1R, is predominantly distributed in the PVN, in which CRH and AVP neurons are located [23]. It has also been reported that OX2R antagonists attenuated the increase in ACTH induced by stress and orexin stimulation, indicating that OX2R is functionally involved in the stress-mediated orexin action of the HPA axis activation in the hypothalamus [24,25]. In the present study, OX2R was found to be expressed in the mouse pituitary tumor cell line AtT20 as well as in human pituitary corticotrope cells, suggesting that OX2R is involved in the control of ACTH, not only in the hypothalamus but also in the pituitary.

Of interest, in the present study, orexin A was found to enhance the expression of CRHR1, leading to an enhancement of CRH signaling by AtT20 cells. The effect of orexin on the expression of CRH receptors has also been reported. For instance, the expression of CRHR1 was increased by repeated stress in rats, and the receptor upregulation was attenuated by the administration of orexin receptor antagonists [26,27]. Those studies suggested that stress-induced orexin stimulation promotes CRHR1 expression, consistent with the results of the present study. Since orexin neurons are also expressed in CRHR1 and have physiological input from CRH [8], there is a very close interaction mediating stress response between orexin and CRH signaling.

Regarding the direct effect of orexin on pituitary corticotropes, Samson and Taylor reported that orexin alone had no effect on ACTH secretion but that, in the presence of CRH, orexin A inhibited CRH-induced ACTH secretion more potently than orexin B in rat pituitary primary cultured cells [28]. Their findings are partially inconsistent with the results of the present study, possibly due to differences in the types of cells used. They suggested that the effect of orexin on corticotropes was mediated by the OX1R-mediated PKC pathway [28], whereas OX2R was predominantly detected by RT-PCR in AtT20 cells in the present study. It should be taken into account that the expression of orexin receptors in pituitary cells has been reported to be varied among animal species [29]. AtT20 cells have been shown to have some differences from native corticotropes in endocrine activity [30], and those differences might be related to the difference between the results of the present and previous studies.

We have been focusing on BMP signaling in various tissues [17]. BMP stimulation is converted into phosphorylation of Smad1, Smad5, and Smad9 (Smad1/5/9), which, together with Smad4, called Co-Smad, translocate to the nucleus and regulate the expression of various genes. Inhibitory Smads, such as Smad6 and Smad7, suppress this phosphorylation and negatively regulate the signal. We have also revealed an interrelationship between orexin and BMP signaling in various endocrine systems. In the pituitary, orexin A suppressed PRL production by inhibiting BMP-4/Smad signaling in rat somatolactotrope GH3 cells [22]. In the ovary, orexin A enhanced FSH-stimulated progesterone production by suppressing BMP-6/Smad signaling in granulosa cells [31]. The common link between those results and the results of the present study was the finding that orexin upregulated inhibitory Smad6/7. A functional link between orexin and inhibitory Smad has also been reported by other laboratories. Orexin A treatment affected the expression of a variety of genes related to cell proliferation and metabolism in HEK293 cells overexpressing orexin receptors [32], in which the BMP-Smad regulation with enhanced Smad7 expression was functionally involved. However, it is still uncertain what intracellular signals of orexin A can modulate the BMP-Smad signaling. We have also reported that melatonin, a circadian hormone, can regulate BMP-Smad signaling via inhibitory Smad6/7 [33]. Considering that BMP-4 and melatonin are mutually linked in the control of clock gene expression in AtT20 cells [34], this novel interaction between BMP and orexin signaling might also be involved in the regulation of the circadian profile of the HPA axis.

## 4. Materials and Methods

### 4.1. Reagents

CRH, Dulbecco’s Modified Eagle’s Medium (DMEM), and DMEM high glucose were purchased from Sigma-Aldrich Corp. (St. Louis, MO, USA). Recombinant human BMP-4 was purchased from R&D Systems Inc. (Minneapolis, MN, USA), and human orexin A was purchased from FUJIFILM Wako Pure Chemical Corp. (Osaka, Japan).

### 4.2. Cell Culture

Mouse corticotrope AtT20/D16v (AtT20) cells under passage 20 were cultured in DMEM supplemented with 10% (*v*/*v*) fetal calf serum (FCS), penicillin and streptomycin in 12-well plates under a 5% (*v*/*v*) CO_2_ atmosphere at 37 °C. Mouse gonadotrope LβT2 cells were cultured in DMEM high glucose supplemented with 10% (*v*/*v*) FCS and antibiotics. After preculture with the growth medium, the medium was changed to a serum-free medium.

### 4.3. RNA Extraction and Quantitative PCR

After preculture, AtT20 cells (1 × 10^5^ viable cells) were transferred to a serum-free medium and treated with orexin A alone, or with a combination of CRH and orexin A for 24 h. In experiments using co-treatment with BMP-4 and orexin A, the cells were transferred to a serum-free medium and treated with orexin A for 24 h, while the treatment time of BMP-4 was set between 1 h and 24 h. The culture medium was then removed, and total cellular RNA was extracted using TRI Reagent^®^ (Cosmo Bio Co., Ltd., Tokyo, Japan). Primer pairs for PCR were selected from different exons of the corresponding genes as follows: mouse OX1R: 1261-1283 and 1660-1682 (BC119583.2); OX2R: 1216-1238 and 1422-1443 (BC140957.1); and BMP type II receptor (BMPRII): 1787-1806 and 1944-1963 (NM_ 007561.4). Mouse POMC, Id-1, Smad6, Smad7, ALK2, ALK3, ALK6, ACTRIIA, and ribosomal protein L19 (RPL19) were selected as we reported previously [21]. The extracted RNA (1 μg) was subjected to an RT reaction using ReverTra Ace^®^ (TOYOBO CO., LTD., Osaka, Japan) with a random hexamer and deoxynucleotide triphosphate (dNTP). After optimizing the annealing conditions for each pair of primers, quantitative PCR was performed to quantify the level of target gene mRNA using the LightCycler 96 System (Roche Diagnostic Co., Tokyo, Japan). The relative expression of each mRNA was determined by the ΔΔCt method, in which ΔCt was the value obtained by subtracting the Ct value of RPL19 mRNA from that of the target mRNA. The amount of target mRNA relative to RPL19 mRNA was expressed as 2^−(ΔΔCt)^, and the results were expressed as the ratio of target mRNA to RPL19 mRNA.

### 4.4. Western Immunoblotting

Cells (1 × 10^5^ viable cells) were precultured with DMEM containing 10% (*v*/*v*) FCS and antibiotics in 12-well plates. After preculture, the medium was changed to a serum-free medium and treated with orexin A for from 24 h to 48 h. In experiments using co-treatment with BMP-4 and orexin A, the cells were stimulated with the indicated concentrations of BMP-4 for 60 min after a 24 h treatment with orexin A. The treated cells were solubilized in 100 μL RIPA lysis buffer (Upstate Biotechnology, Lake Placid, NY, USA) containing 1 mM Na_3_VO_4_, 1 mM NaF, 2% (*v*/*v*) SDS, and 4% (*v*/*v*) β-mercaptoethanol. Western blot analysis was performed using the cell lysates with specific antibodies against phospho-Smad1/5/9 (pSmad1/5/9; Cell Signaling Technology, Inc., Beverly, MA, USA; catalog #13820), total-Smad1 (tSmad1; Cell Signaling Technology; catalog #6944), CRH receptor type-1 (CRHR1; Aviva Systems Biology, San Diego, CA, USA; catalog #OASG01837), Smad6 (Cell Signaling Technology; catalog #9519), Smad7 (R&D Systems; catalog #MAB2029) and actin (Sigma-Aldrich Co. Ltd., St. Louis, MO, USA; catalog #A2066). The blotted bands were analyzed by the C-DiGit^®^ Blot Scanner System (LI-COR Biosciences, Lincoln, NE, USA) by scanning the integrated signal intensities. To evaluate the phosphorylated Smad contents, the ratios of the digitized levels of pSmad/tSmad bands were calculated. For CRHR1, Smad6, and Smad7 protein levels, the ratios of the digitized levels of each actin band were calculated.

### 4.5. Statistical Analysis

Experimental results are shown as means ±SEM of data from at least three independent experiments, each performed with triplicate samples. The data were then subjected to ANOVA followed by the Tukey post hoc test or unpaired *t*-test. All statistical analyses were performed using EZR Ver 1.42 (Saitama Medical Center, Jichi Medical University, Saitama, Japan) and the interface for R Ver 4.0.0 (The R Foundation for Statistical Computing, Vienna, Austria). *p* values < 0.05 were accepted as statistically significant.

## 5. Conclusions

Collectively, our results showed that orexin modulated POMC transcription by enhancing the expression of CRH receptors as well as suppressing BMP-Smad signaling. Our results suggest that orexin plays a modulatory role in the secretion of ACTH from the anterior pituitary. From a clinical point of view, orexin receptor antagonists, such as suvorexant and lemborexant, currently used in the treatment of insomnia, have been reported to decrease ACTH in rats and humans [35,36]. Control of orexin signaling might be a new strategy for stress management and/or treatment of Cushing’s disease by inhibiting POMC transcription in corticotropes.

## Data Availability

Data is contained within the article.

## Abbreviations

ACTH	adrenocorticotropin
ActRII	activin type II receptor
ALK	activin receptor-like kinase
AVP	arginine vasopressin
BMP	bone morphogenetic protein
BMPRII	BMP type II receptor
CRH	corticotropin-releasing hormone
CRHR	CRH receptor
HPA	hypothalamic-pituitary-adrenal
ORX	orexin A
OX1R	orexin type 1 receptor
OX2R	orexin type 2 receptor
POMC	pro-opiomelanocortin
PVN	paraventricular nucleus
(TGF)β	transforming growth factor (TGF)β
